# Immunochromatography Test Kit for Paralytic Shellfish Toxins (PSTs) and Transition of PSTs in Scallops

**DOI:** 10.14252/foodsafetyfscj.D-25-00017

**Published:** 2026-02-12

**Authors:** Ryoji Matsushima, Yusuke Shibahara, Shinnosuke Kaga, Hiroshi Oikawa, Satoshi Numano, Ryuichi Watanabe, Hajime Uchida, Mayu Ozawa, Toshiyuki Suzuki

**Affiliations:** 1 Fisheries Technology Institute, Japan Fisheries Research and Education Agency, 2-12-4, Fukuura,Kanazawa-ku, Yokohama, Kanagawa 236-8648, Japan; 2 Shimadzu Diagnostics Corporation, Ueno Frontier Tower 20F, 3‑24‑6 Ueno, Taito‑ku, Tokyo 110‑8736, Japan; 3 Iwate Fisheries Technology Center, 3-75-3, Heita, Kamaishi, Iwate 026-0001, Japan; 4 School of Marine Biosciences, Kitasato University, 1-15-1, Kitazato, Minami-ku, Sagamihara, Kanagawa252-0373, Japan

**Keywords:** paralytic shellfish toxins, scallops, immunochromatography, antibody, PSTs

## Abstract

MT test Immunochromato-PSP had been developed in a collaborative research project. In this kit, the previously developed mouse monoclonal antibody GT-13A designed against GTX2/3 is used. Since STX and its analogs (STXs) are small molecules, a competitive inhibition format with modified-STX is applied. The formation of Avidin Biotin complexes to trap modified-STX on the test line showed interference by the bivalve matrix, so we improved the kit with oligonucleotides trapping complementary strands. The affinity of the GT-13 antibody differs depending on the STX analogs present and does not correspond to relative toxicity. Therefore, it is necessary to accumulate data in advance on paralytic shellfish toxins (PSTs) toxin profiles for the local target species and area. Since this kit is intended to be used in screening, it is necessary to consider a dilution factor that will never lead to a false negative against the regulatory value. Although this kit is qualitative, it can be recorded and compared objectively as semi-quantitative data by imaging and quantifying. It can also be used to determine PSTs presence in seawater samples. In recent years, the problem of PSTs has become more serious in the east and north of Japan. We are considering using the kit for monitoring scallops in one prefecture and have confirmed that some of the samples could be assessed with the kit and applied to screening. However, we also observed transformation of PSTs after the shellfish became highly toxic, limiting the utility of the kit in these cases.

## Introduction

In Japan, shellfish toxin problems with bivalve mollusks are mainly caused by diarrhetic toxins (DSTs) and paralytic shellfish toxins (PSTs). Based on the Food Sanitation Act, the government has established regulatory limits for PSTs and DSTs. The monitoring and inspection systems for shellfish toxins have been established to prevent food poisoning caused by these toxins, thereby ensuring the safety of shellfish. The PSTs tests are performed using the mouse bioassay (MBA) in accordance with the AOAC Official Method 959.08^1^^)^. On the other hand, in recent years, there has been a global trend toward using alternative methods to animal testing due to growing social concern in animal welfare. In Japan, the Act on Welfare and Management of Animals (Animal Welfare and Management Act) was revised in 2005 to explicitly state the 3Rs international principles for animal experimentation (Replacement: use of alternative methods, Reduction: reduction in the number of animals used, Refinement: reduction of pain and suffering of experimental animals). The Ministry of Agriculture, Forestry and Fisheries has issued a notice on “Monitoring and control measures for shellfish poisoning in marine production areas”^2^^)^ and has also established new “Guidelines for risk management of shellfish poisoning in bivalve mollusks, etc”^3^^)^, which have made it possible for PSTs to introduce instrumental analysis testing and screening methods using simplified analytical methods for risk management in marine production areas.

## Development of Immunochromatography Kit for PSTs

**Fig. 1.**
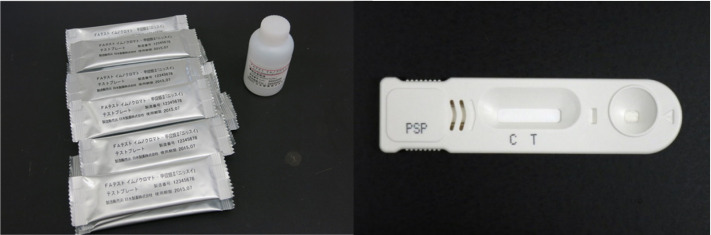
MT test Immunochromato-PSP. Each test plate is individually packaged and comes with a sample dilution solution bottle.

Immunochromatography methods, such as lateral flow immunochemical assay, do not require special equipment and allows for visual determination in a short time. Due to its speed and simplicity, it is expected to be widely used as a preliminary screening method prior to ELISA or instrumental analysis, as well as an analytical method in field work. Our group developed a simple test kit for PSTs based on the immunochromatography method under the research project on Regulatory research projects (Fig. 1).

**Fig. 2.**
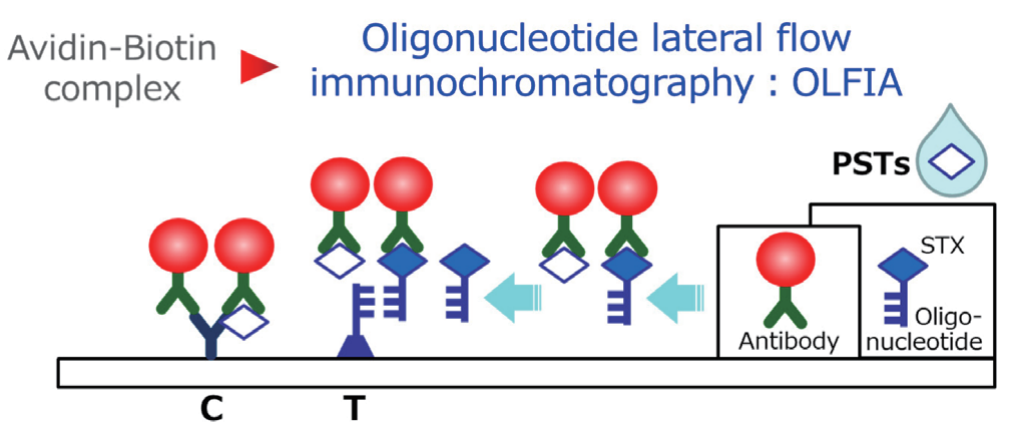
Composition and principle of this immunochromatography kit. White diamonds represent PSTs. Blue diamond-key shapes represent oligonucleotide-labeled STX. Navy trapezoid-key shapes represent protein-binding oligonucleotides. Red ball-Y represent gold colloid conjugated anti-PST antibody.

This kit allows visual evaluation within 20 minutes by diluting the bivalve extract obtained in the same manner as in the MBA with the included dilution solution at an appropriate ratio and adding it on a test plate. This kit uses the previously developed monoclonal antibody against Gonyautoxin (GTX) 2/3 developed by the Osaka Institute of Public Health^4^^)^. Because PSTs molecules are small (approximately 300 g/mol), the presence of PSTs is detected by competitive inhibition ELISA. The PSTs in the sample migrate to the left while mixing with the competing reagent modified STX and gold colloid-bound antibody (Fig. 2). The gold colloid antibody bound to the competitive reagent is trapped by the protein-binding oligonucleotide immobilized on the kit plate, forming a line at T. If there are sufficient quantities of PSTs in the sample, the colloidal gold antibody will trap the PSTs, and no line will appear at T. The gold colloid antibodies that migrate further are trapped by the immobilized anti-mouse antibody and form a line at C.

**Fig. 3.**
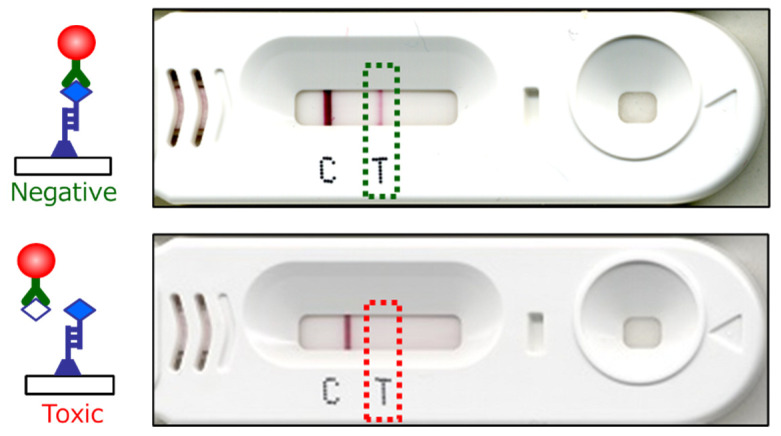
Immunochromatography kit evaluation of sample. The upper is the result of a negative sample, in which the T line appears clearly. The bottom is the result of a toxic sample, in which the T line does not appear. This is the opposite reaction to that of a general immunochromatography kit.

Due to the competitive method of detection, the test line (T) becomes faint or invisible in toxic samples, while the test line (T) appears clearly visible in non-toxic samples (Figs. 2,3).

Initially, we considered a method for coloring the test line (T) by capturing the complex of gold colloid-bound antibody and competitive reagent (Biotin-STX)^5^^)^ using the avidin-biotin interaction. However, some bivalve species showed matrix inhibition of the reaction; we improved the method to capture the complex by the complementary strands of oligonucleotides (Oligonucleotide lateral flow immunochromatography: OLFIA method) (Fig. 2). This made it possible to easily detect PSTs in major shellfish produced domestically in Japan. The antibody used in the kit is mouse monoclonal antibodies, but its reactivity to each component of PSTs varies depending on the component, and the sensitivity to the actual PST component is determined by competition with modified STX. The antibody has a high affinity for the antigen GTX 2/3 as well as the weak toxin C1/2 (approximately twice the value of EC_50_ of GTX2/3)^4^^)^, its affinity does not correspond to the toxicity. Therefore, it is necessary to accumulate data with the PST toxin profiles of target samples and areas, correlation between the MBA and the test kit. To use the test kit, the same extract as the MBA solution is diluted to a specified ratio and 100 μL is added to the test well. This kit is intended for use in screening, and it is necessary to consider an appropriate dilution ratio that will never lead false negatives relative to regulatory values. Due to the influence of the sample matrix and low pH, the dilution is generally 30 times or more.

## Determining Sample Dilution Ratio and Usage of the Kit

**Fig. 4.**
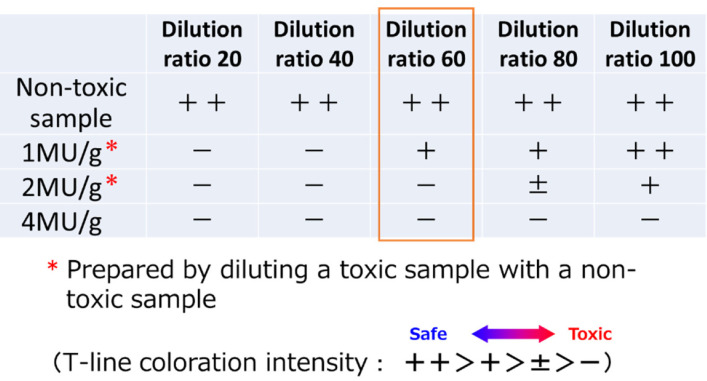
Schematic diagram of the MU/g sample sets and the results depending on the dilution ratio.

First, a series of samples adjusted to 1–4 MU/g are prepared by diluting the extract of a highly toxic sample confirmed by MBA with the extract of a non-toxic sample of the same species. Apply a series of dilution factors to this series of MU/g samples, and the appearance of the T line is checked to determine the desired screening value and dilution factor. For normal samples, screening of 2–3 MU/g is possible at about 60-80 times dilution. However, in areas where plankton containing a lot of C toxins such as *Alexandrium pacificum* and *Gynodinium catenatum* occurs, the dilution ratio may be more than 100 times. Also, the dilution ratio is low for samples containing a lot of GTX1/4 or neoSTX which have relatively low sensitivity. In this case, 2 MU/g can be screened with a 60-fold dilution (Fig. 4).

This kit can also be applied to seawater samples with toxic plankton. The plankton in seawater samples are counted and concentrated. After extraction with 0.2 M acetic acid, the samples are diluted and applied to the kit. By diluting according to the alert or attention density of specific algae, it is possible to confirm the occurrence of toxic species without direct survey using a microscope and without any knowledge of dinoflagellates. Although this kit is qualitative character, by imaging and quantifying the T line, the results can be recorded as semi-quantitative data and compared objectively. The test plate image can be digitized using other than dedicated devices like digital camera or CCD scanner and then quantified using commercial image analysis software or freeware like ImageJ. The C/T value, calculated by dividing the C line value by the T line value, corresponds to the strength of the toxin, being larger when the toxicity is high and smaller when the toxicity is low. In addition, the C/T value has a higher correlation coefficient than the T line value alone, making it a more stable index. On the other hand, the T/C value, obtained by dividing T line value by C line value, is the reciprocal of the C/T value, and is inversely proportional to toxicity, with a lower correlation coefficient than the T line value alone.

## An Example of PSTs Verification with The Kit in Northern Area of Japan

**Fig. 5.**
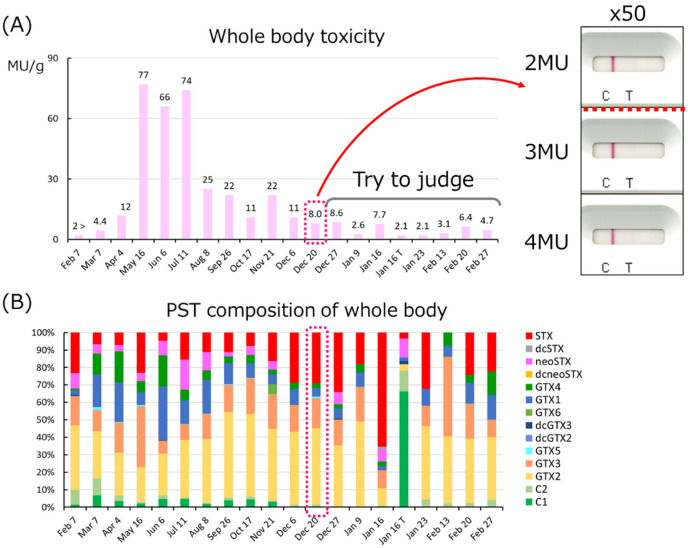
Trends of PSTs in scallops from 2022 Feb to 2023 Feb. The graph above (A) shows mouse toxicity of MBA (MU/g), and the graph below (B) shows the percentage of the PSTs component analyzed by LC/MS/MS from same fixed-point samples except Jan16T. Jan16T is a sample from a different point.

In recent years, the problem of PSTs has increased as toxicities of bivalves have become higher and more prolonged in the northern area of Japan. We are considering using the kit for monitoring scallops in one prefecture and have observed the transformation of PSTs after they become highly toxic. The variation of PSTs in the whole body is shown in Fig. 5.

**Fig. 6.**
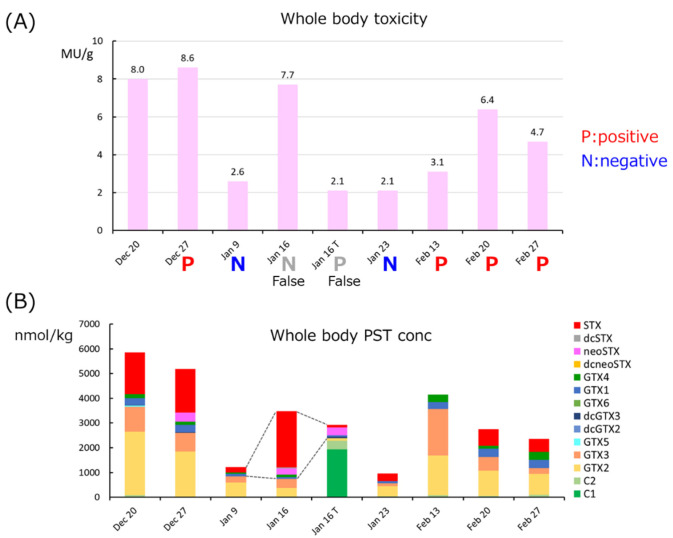
Toxicity and PSTs composition of scallops Dec20-Feb27. The graph above (A) shows 2MU/g screening results and mouse toxicity of MBA (MU/g) in the whole body. The graph below (B) shows the concentration of PSTs analyzed by LC/MS/MS in the whole body.

Between February 2022 and February 2023 the toxicity increased from March, reached a peak in May, and then decreased. During the period of rise, the percentage of GTX1/4 is high, but during the decline, the percentage of GTX2/3 increases, and even higher percentage of STX in the second half of the year. When the dilution ratio for use with the immunochromatography kit was tested using a sample of 8 MU/g taken on 20th December, it was possible to distinguish 3MU/g or higher at 50 times the dilution ratio. The whole body samples taken after 27th December were judged with a 50-fold dilution. The results were generally correct, but some samples were judged incorrectly (Fig. 6).

The false negative Jan16 had many highly toxic but low sensitivity components (STX and neoSTX), while the false positive Jan16T had a very high content of the weak toxic C1/2, from the site was affected by the specific *Alexandrium* sp. bloom. Immunochromatography kit reactions are affected by changes in PSTs composition, so they are not necessarily suitable for situations where the composition of the PSTs changes due to long-term toxification, and they cannot be applied in the same way at every location.

## Summary and Conclusions

The main problems with shellfish toxins in Japan are the DSTs and PSTs. Guidelines are established by Authorities and safety of shellfish is well managed. Guidelines recognize the use of simple kits as an alternative measure, and an immunochromatography kit for PSTs was developed. The principle of kit is competitive inhibition, and by appropriately defining dilution factor, it can be utilized for screening of PSTs. Considering the use of this kit in areas where PSTs contamination is serious, there were issues in cases of high toxicity scallops or different compositions of plankton.
